# Non-medical prescribing of chemotherapy: engaging stakeholders to maximise success?

**DOI:** 10.3332/ecancer.2014.417

**Published:** 2014-04-10

**Authors:** Elaine Lennan

**Affiliations:** University Hospital Southampton, Hampshire SO16 6YD, United Kingdom

**Keywords:** non-medical prescribing, chemotherapy, nurse-led clinic

## Abstract

**Aim:**

This study report examines the views and experiences of professional stakeholders about non-medical prescribing (NMP) of chemotherapy.

**Background:**

The introduction of open formulary NMP has created opportunities to radically change health-care delivery. For chemotherapy services, the most recent advice from the National Chemotherapy Advisory Group [Department of Health (2009) Chemotherapy Services in England, ensuring quality and safety: a report from the National Chemotherapy Advisory Group, London Her Majesty’s Stationary Office] clearly endorses the development of nurse- or pharmacist-led chemotherapy clinics. This is very much welcomed but is based on very limited evidence as to their effectiveness.

**Design:**

A fourth-generation evaluation study.

**Methods:**

A purposeful sample of 23 stakeholders connected with the chemotherapy service was used. A serial data collection technique with individual interviews followed by uni-professional focus groups was adopted. Finally, a multi-professional focus group was held to determine the strategic way forward. Data were collected in 2009–2010.

**Results:**

The study illuminated the key features necessary to maximise success of NMP in chemotherapy clinics and captures the importance of good working relationships. Whilst different practice models will emerge, fundamental and core to services is the need for good team working, established and effective communication strategies, and most importantly avoiding isolation in practice. This study additionally reinforced any evaluation takes place within preexisting political contexts and in particular medical dominance. Not all medical colleagues agreed with or wanted NMP for their patients, highlighting difficulties of developing new models of working within a resisting culture.

**Conclusion:**

No objections to NMP of chemotherapy were found, but, clearly, the context of practice needs to be agreed and supportedby all professional stakeholders.

**What is already known about this topic**
- Open formulary non-medical prescribing has been rapidly introduced over the past decade.
- Little research has been conducted in acute care and none in the chemotherapy setting.
- Cancer policy recommends the introduction of nurse-led chemotherapy clinics.

**What this paper adds**
- Non-medical prescribing (NMP) in chemotherapy is appropriate with the right model of practice.
- Well-established professional relationships are a key to success.
- NMP is not appropriate in isolation of the multidisciplinary team (MDT).

**Implications for practice and/or policy**
- Nurses need to demonstrate the value of non-medical prescribing in chemotherapy using available metrics.
- Models of practice need to ensure good communication channels, MDT working, and transparency of prescribing.

## Introduction

The introduction of NMP, that is, the prescribing of medicines by health-care professionals other than doctors, was first mooted in the United Kingdom (UK) in 1986 [1]. It is now an exciting opportunity for nurses and others, made possible by recent government policy [2]–[5].

The beginning of the NMP movement saw several problems in implementation and acceptance. First, in the eagerness to meet short-term training targets, strategic planning regarding the implementation and integration of NMP was overlooked [6]–[9].

Second, the rapid changes in legislation unnerved some within the medical profession, creating a climate of scepticism and uncertainty about roles, which, in turn, created a barrier [8–17]. Indeed, Hawkes [18] states the British Medical Association in 2005 reacted in horror and voted solidly for the ‘slowest possible progress’. This is, perhaps, understandable, given that in only six years from the first review, legislative changes had occurred to allow nurses and pharmacists to practice as prescribers [4, 19–23]. Finally, the far-reaching scope of the NMP legislation in England then exceeded anywhere else in the world causing some concern, and whilst nurses and pharmacists were the start, other allied professions had plans to expand their professional scope [5, 8, 10]. Avery *et al* stated

‘The exclusion of doctors from any planning process made it inevitable that nurses would have difficulty implementing this newly acquired skill’ [8, p. 111].

This early concern has been further demonstrated by Latter *et al* [24] and Stenner *et al* [25] with concerns raised around loose cannons of prescribing nurses and a lack of confidence in the preparation of nurses to prescribe.

‘The worry is that they (nurses) have the whole BNF (British National Formulary) to prescribe from. Our practice nurses wouldn’t prescribe controlled drugs for terminal care because they haven’t got the experience to do that, although on paper they could. I think that that is the sort of worry that the general masses [medical profession as a whole] might feel. You are going to get ‘loose canons’, people prescribing too much’ [25, p. 855].

The current legislation allows two forms of NMP known as independent nurse prescribing and supplementary prescribing. Table 1 distinguishes the two [4].

## The context of prescribing in the chemotherapy setting

Chemotherapy services have been fortunate over the past decade to have clear DOH policy to guide services. This is much needed, given the huge rise in demand for chemotherapy due to the following reasons:

(a) early diagnosis;
(b) better outcomes of therapy;
(c) availability of further therapy;
(d) new drugs;
(e) new technologies.

Given this expediential rise, chemotherapy services have struggled with the provision. Chemotherapy services are overstretched, with long waiting times and inefficient services. Policy documents addressed this challenge by clearly outlining new roles for various health professionals and urged a critical examination of the workforce [26, 27]. However, for chemotherapy services, the lack of prescriptive authority for non-doctors hampered some progress.

The most recent directive from the National Chemotherapy Advisory Group (NCAG) [5] was written in response to safety concerns in chemotherapy due to overcrowding and inadequate expertise at various points on the chemotherapy pathway. Amongst many other recommendations, NCAG clearly endorses the development of nurse-led chemotherapy clinics, and this is a considered and welcome acknowledgment of the chemotherapy nursing workforce. It is, though, without an evidence base to support its effectiveness.

Nurse-led clinics are not new and have been proven to be of equal or better quality than the medical-led service in many areas [28–34], but for chemotherapy nurses, the inability to prescribe has always been a major source of frustration [35]. Indeed, some chemotherapy clinics exist where the nurse will assess the patient, make decisions about treatment, including alterations and additional drugs, but then wait for the doctor to prescribe. This is little more than a prescription-writing exercise, as the nurse has made all the decisions. This practice confuses accountability and potentially hides the contribution of the nurse.

The recent changes in prescriptive legislation now allow some, not all, chemotherapy nurses to implement the initiatives of recent government policy [25]. Fitzsimmons *et al* [35] cautioned chemotherapy nurses to be clear not to ‘just substitute’ for the doctor in developing new services. There is an opportunity to add value of nurses and nursing to the previous medical task, and this was a clear premise for developing this study. NMP now paves the way for the development of robust services on which to develop further research.

## Literature review

Given the time frame and pace of change, it is not surprising to note there were only a small number of empirical papers examining the subject prior to this study. Most were based in primary care and predominantly examined community nurse prescribing [24, 36–41].

Generally, NMP is viewed as positive, though some organisational and process issues hampered practice. The transferability of these study findings to chemotherapy is limited to the process and professional aspects of NMP, not necessarily the practice setting. Chemotherapy treatments are extremely complex. Traditional chemotherapeutic agents act by killing cells, and as a treatment modality can be lethal, affecting every system of the body. It is simply not possible to compare with other services.

### The acute setting

Chemotherapy services are currently situated within acute care, and, therefore, it seems appropriate to consider whether evidence exists in this context. Buckley *et al* [15] examined NMP from a pharmacist perspective in acute care. Support for the concept was found and highlighted inter- and intra-professional relationships as key to success. Medical staff had reservations, and though not objecting, wanted strict controls on practice, in essence, a restricted formulary. Nurses felt pharmacists needed better contact with patients, whilst pharmacists thought nurses needed better pharmacology knowledge, not an unsurprising finding. However, an interesting finding is that of boundaries. The views expressed highlighted a possible competition between nurses and pharmacists for the niche to be able to extend their prescribing roles. This clearly suggests that the involvement of all professions at the concept and strategy development stage could help to determine the appropriate professional for that role, getting the right person in the right place at the right time. Fitzpatrick [42] concurs in a study with in-patients, where broad support for pharmacist prescribing was found, but general awareness about who could and could not prescribe was lacking. Those who were unsupportive of the concept felt they might change their view as they became more exposed to pharmacy prescribing practice. Both these studies support the idea that the inclusive nature, as in this study, of engaging all health-care professionals will be beneficial.

Goswell and Siefers [43] and Crew [44] describe their own practice in an acute ward settings and conclude that they currently prescribe more than their counterparts in medicine and offer enhanced patient care through safe and timely access to medicines, increased patient involvement in decisions, and collaborative team working. They further stress prescribing never occurs in isolation but use the MDT for support. Jones *et al* [45] recognising the lack of research in acute care developed an evaluative case study. Findings show an equivalence between doctors and prescribing nurses, and likewise Poonawala *et al* [46] demonstrated enhanced practice without compromising safety in the ITU setting.

### Cancer and chemotherapy

Two papers relate to the cancer setting. A national survey of Macmillan nurses investigated their views in relation to nurse prescribing [47]. A distinct reluctance to prescribe was found, with reasons given as lack of mentorship, insufficient focus, and depth of training and organizational constraints. Fitzsimmons *et al* [35] is the only study prior to this study to examine the chemotherapy setting in relation to nurse-led care. Users had firm views on traditional medical and nursing roles and in reflecting on their care found great difficulty in conceptualizing how changing these roles might enhance their care. Some expressed that they would be quite satisfied with nurse-led care; others said ‘as long as the doctor was in the background’. Others stated that they would not be happy.

Professionals held mixed views. Medical staff said that it was a useful development, whilst others, mainly nurses, felt there would be a loss of the nursing role. A major finding of this study was the use of the term doctor–nurse substitution. Participants envisaged that nurses would be working in relative isolation, making sole decisions about treatment plans rather than working in separate but complementary roles as part of the MDT.

## This study

To understand the complexities of the NMP in chemotherapy clinics, this study asked the question, what are the views and experiences of professional stakeholders about NMP practices within a chemotherapy clinic? It was imperative to study the concept of NMP in the acute setting not only to add to the whole body of knowledge regarding NMP, but also to identify whether the many advantages and barriers within the literature in primary care are context-specific.

### Aim

The study was based in one chemotherapy unit and used a dialectic process to explore stakeholder’s views and experiences of practice. Once these views were known, the aim was to use these views to negotiate a strategic vision for the future development of NMP within the chemotherapy service. The dynamic nature of the study meant that each stage built on the next. The specific objectives of the study are as follows:

(a) to identify all professional stakeholders involved in NMP within a local chemotherapy service;
(b) to provide a local perspective on NMP of chemotherapy including the range of prescribing practices currently in operation and factors that facilitate or inhibit effective prescribing in practice;
(c) to elicit from professionals, their claims, concerns and issues in relation to NMP of chemotherapy, first as seen from an individual stakeholder perspective;
(d) to use the evaluation of the individual perceptions and experiences as a basis to seek either agreement or rejection on a collective basis with other stakeholders of the same professional group;
(e) to attempt to reach a consensus of opinion across the professional groups by further actively engaging all stakeholders in a structured yet flexible forum of focussed debate of the issues that emerge from the evaluation;
(f) by means of a continuous dialectic engagement and by being responsive to the findings of the evaluation, to attempt to negotiate a strategic vision for the further development of NMP in the local chemotherapy setting;
(g) from the agreed strategic vision, to develop an action plan for implementing changes to local practice, rooted in a construct of reality and agreed by all stakeholders of the service.

## Study Design

### Fourth-generation methodology

The inclusive nature of engaging all stakeholders in the development of the service was central to the study, as the research framework attempted to develop a future strategic direction. Given this stance, it was important to consider an appropriate and effective evaluation methodology that reflected and captured all the viewpoints and additionally offered a change process. Fourth-generation evaluation (FGE) or constructivist evaluation is one such methodology that has been growing in popularity in recent years, as the method not only demands an equal voice for all to evaluate the service, the process itself acts as a catalyst for change [48, 49].

The outcome of an FGE is not about bold outcomes of the way things are but instead represent meaningful constructions that participants form in trying to make sense of where they find themselves [50]. The outcomes are not facts but are constructions created through the interactive dialogue of all stakeholders. This hermeneutic approach accepts that participants are self interpreting and bring their construction to the negotiating table [51, 52]. Hermeneutics is the theory of text interpretation, written verbal, and non-verbal communication, and is the basic underpinning theory of this study [52]. However, this is further developed by a concept of double hermenteutics with involves not only interpreting and understanding text, it considers what people do, how people understand their world, and how that understanding shapes their practice. People can think, make choices, and use new information to revise their understandings, and then use the new knowledge and insights to change their practice [53].

Within this philosophy, stakeholders are the creators of knowledge, and dialogue between users, providers, and researchers is ongoing [54]. It is the claims (favorable stance), concerns (unfavorable stance), and issues (disagreement between reasonable people) of those stakeholders that serve as the focus for this research study.

### The flow of FGE

The negotiation process began with semi-structured individual interviews of the stakeholders and moved to further engagement with the stakeholders in focus groups. Whilst it is recognized that stakeholder groups may have had very different views, the purpose of negotiation is to welcome stakeholders as partners in every aspect of the design, implementation, interpretation, and resulting actions of the developing service [50, 55].

**Figure 1.**
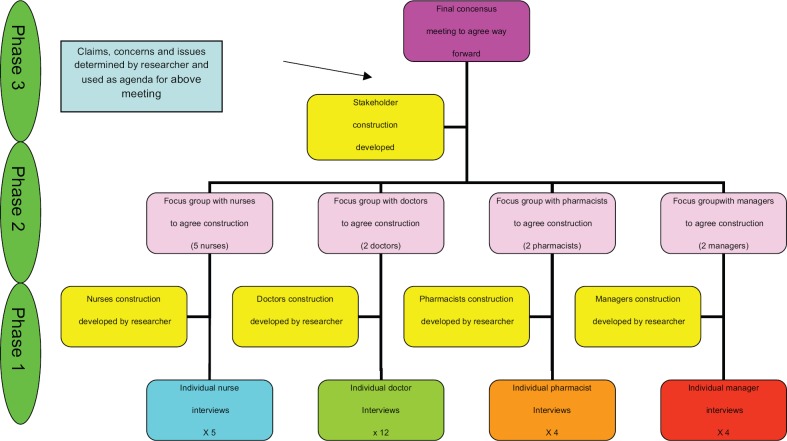
A framework for engaging with stakeholders.

The study had a three-phase approach, as shown in Figure 1. The views from the individual interviews were taken to the uni-professional focus group and used for debate. This forum was to try to get a consensus amongst that professional group. Once this was complete, the views of the uni-professional groups were used to debate at a multi-professional forum. The aim here was to try to get an agreed view and strategic direction for the organization.

### Study setting

The setting for the study was the chemotherapy clinic at the local cancer centre. Patients receive chemotherapy on a cyclical basis the timing of which is different for each cancer or treatment. Frequently, this means returning to the hospital for an assessment prior to receiving the chemotherapy the following day. If deemed fit, the health professional prescribes the treatment and any supportive medicines in order for the pharmacy to prepare the drugs. This setting was the busiest centre in the region treating all cancers. The annual attendances are 7500+, with an expected 12% increase each year. Given such numbers, innovations such as NMP are likely to have a major impact on this service and once robustly established had the potential to be rolled out to the smaller local cancer units. It was the only chemotherapy clinic in the region that had an NMP service and one of the first nationally. The service was not universally using a nurse-led service, and the unit was struggling to expand further. This inconsistency made the ideal setting for this research study.

### Sampling

Each phase had a sampling strategy. As this study was context specific, stakeholders were initially determined by the researcher using the unique knowledge afforded to a researcher from being on the inside and confirmed through discussion with individual stakeholders [56]. The identification of stakeholders included assessing those groups who could potentially benefit, be a victim of or be disenfranchised as a result of this evaluation [55, 57]. Whilst it is absolutely recognized that patients are clear stakeholders in any health service, a conscious and pragmatic decision was taken not to include this group in this study. The focus of this research was health professionals’ views and experiences and engagement with patients regarding any proposals that result from the study will be conducted outside the framework of this research. It is important to justify exclusion of patients at this point, as the current context of health-care service improvement insists on such engagement. However, the focus of this particular study was on understanding professional views to try to gain a baseline to move the service forward. In addition, Fitzsimmons *et al*’s [35] study clearly demonstrated the difficulty in engaging with patients who did not understand the implications of a particular model of care. Participants in this study found it difficult to conceptualize a different system to the one to which they were exposed.

**Figure 2.**
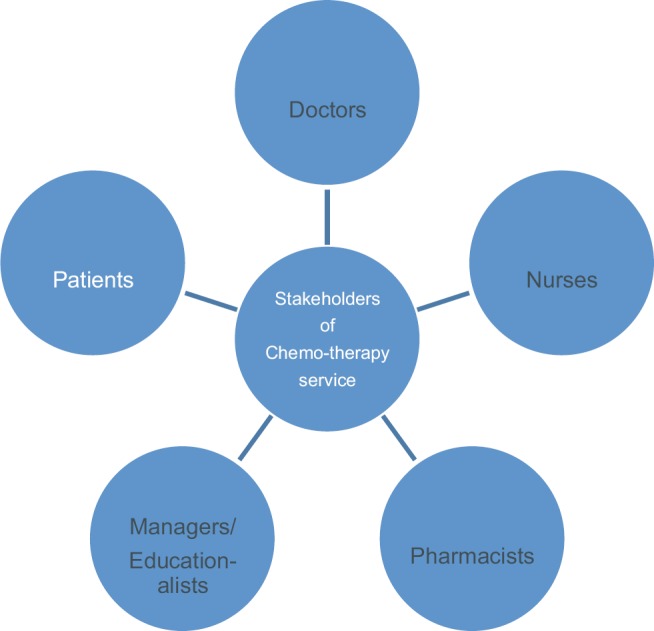
The identification of stakeholder groups.

An initial identification of stakeholders can be observed in Figure 2 and Table 3.

### Exclusion criteria

There were no specific exclusion criteria. Identified stakeholders could refuse to take part or could withdraw at any time.

### Data collection and development of constructions

Several data collection techniques were used within this study and are summarized in Table 2. FGE is built upon the view that discursive processes should predominate. Given that the aim of the study was to elicit views and experiences of stakeholders, it was decided a semi-structured interview approach was the most appropriate for phase 1 of the study. This would allow focus on the subject but additionally permit exploration of experiences as they arise [56, 58]. Stopping data collection following the interviews would prevent the earliest respondents from reacting, challenging, or agreeing with the construction. This would clearly impede ownership of the construction, a key aspect of negotiation [59]. It was therefore decided that following the individual interview, a focus group with each stakeholder group would be conducted being phase 2 of the study, as a way to enhance the dialectic process. The aim of this meeting was to agree the construction through negotiation. Finally, phase 3 involved a further multi-professional focus group to negotiate a way forward. This included finding areas of common ground and negotiating areas of disagreement.

### Ethical considerations

The key ethical considerations for this study were confidentiality and data protection. Mindful of these concerns, on approaching the Local Research Ethics Committee for approval, these issues were open and transparent. Full approval was given.

### Data analysis

The purpose of data analysis is to provide structure to, and elicit meaning from, research data (Table 4). Data analysis in FGE inherently takes a structured path. All data from phase 1 interviews were analysed as soon as possible after the event to allow for probing of issues raised with future participants [51]. Transcripts of interviews were examined to inform the study of possible themes using a systematic qualitative data management package known as NUDIST [50].

As views were unknown at the beginning of data collection, a sensible approach was taken to interview the first participant based solely on availability. The method involved hermeneutic dialectic negotiation [48, 50, 51]. Hermeneutic relates to its interpretive character, and it is dialectic, as it represents a comparison and contrast of divergent views with a view to achieving a higher-level synthesis [48]. The aim of the process was to form a connection between all viewpoints or constructions that allows mutual exploration by all parties.

**Figure 3.**
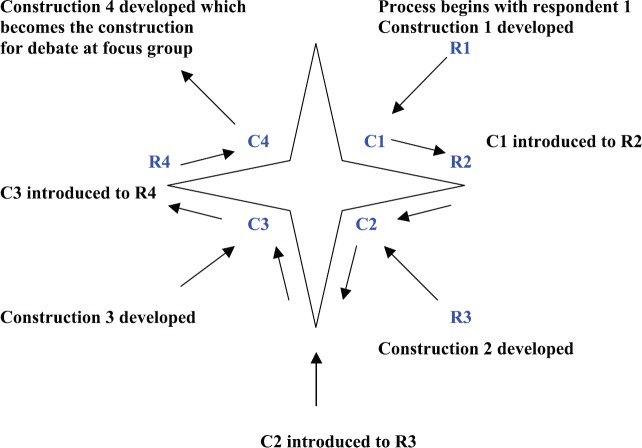
The progress through the hermeneutic dialectic circle for a stakeholder group. The final construction emerges, and the circle is complete. The circle should continue until all available participants have been included.

The first interviewee or respondent determined the initial construction or initial viewpoint on NMP and so began a hermeneutic circle. The respondent was asked to describe and evaluate the subject as he/she saw it. This was a personal viewpoint and included observations, concerns, likes, and dislikes. The central themes, concepts, ideas, values, concerns, and issues from this viewpoint were developed by the researcher into an initial formulation known as construction 1. Construction 1 was completed prior to interview 2 in order to be able to introduce the findings from construction 1 to respondent 2 [50]. This was achieved in all cases. Respondent 2 was given the same freedom of expression as respondent 1, but when no new information was forth coming, the themes from construction 1 were introduced. This allowed respondent 2 to comment on respondent 1’s thoughts. As a result, respondent 2 produced information from a personal viewpoint but was also able to critique respondent 1. The analysis of respondent 2 became construction 2, which is a hybrid of respondent 1 and 2, and continues the development of the circle. This process continued with all stakeholders (i.e., four in the case of pharmacists) until the circle is complete. This is shown in Figure 3.

Construction 4 was the basis for discussion at the pharmacists focus group, where claims and concerns were agreed and issues discussed in an attempt to resolve any differing opinion. The result is the collective pharmacists viewpoint or construction. This process was repeated for all professional groups. Once the constructions of the professional groups had been agreed, a final consensus meeting was required with all professions to continue the negotiation process.

### Validity and reliability

Trustworthiness or methodological soundness was important to this study. Trustworthiness was established based on the framework presented by Lincoln and Guba [50], being criteria of credibility, transferability, dependability, and confirmability.

## Results

**Figure 4.**
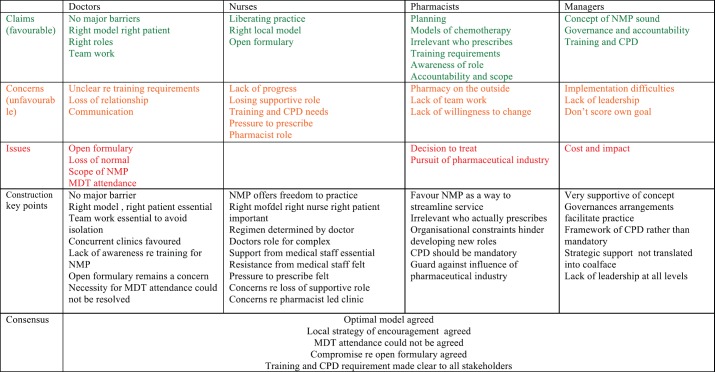
A summary of the individual claims concerns and issues of all the professional groups including the agreed construction and outcome at concensus meeting.

Figure 4 shows a summary of all the participants at each stage, and Figure 3 shows a summary of the developing construction. The results from the concensus meeting multidisciplinary focus group are discussed below.

## Consensus meeting—the way forward

The findings of the study reassuringly revealed much common ground but also some clear differences of opinion. It was encouraging and, perhaps, surprising given the inconsistency in practice to note that no major objection to the concept of NMP existed from the medical staff, but all felt the right model was important.

### The optimum model

In terms of the optimal model for chemotherapy services, all agreed that an alternative cycle model concurrent with the medical team was the preferred option. It was unacceptable to the nurses to develop a model, where the doctors prescribed the treatment but the nurses assessed suitability to receive the treatment. Nurses wanted the full autonomy of NMP. This proposal means the patient would alternate between the doctor and the nurse for each visit for chemotherapy, i.e., cycle 1 nurse, cycle 2 doctor, cycle 3 nurse, etc. Working concurrently to the doctor offered support for the nurse, a sense of staying in control /in touch with patients for the doctors and the ability to offer a shared arrangement for the patient. Indeed, one doctor said

It’s important we don’t go backwards. The last few years have changed chemotherapy services in that it is now very much consultant led, when I first qualified it was the SHOs that did all the chemo and the consultant wouldn’t have a clue. I am all in favour of specialist nurse services especially in chemotherapy but there must be good communication.

Whilst this model was agreed through this dialogue process, it was clearly noted that those present could not dictate practice to those not present.

*Whilst we agree this model you’re gonna have to go and agree the individual practice with each speciality. I can have a view on how others practice but I cannot make it happen. And of course we’re all interested we’ve all turned up to discuss it!*—Doctor.

And also

*What we need is a local champion, doctor, I mean, to support us*—Nurse.

This need for a coalface champion was highlighted in the managers construction, where there was also a concern of ‘scoring an own goal’ by raising issues where none existed. Whilst it was fairly straightforward to get executive support for NMP, it was thought inappropriate to exercise this high authority in insisting practice models change. A better strategy was to win over medical consultants by demonstrating the value of NMP to their service and by engaging them in the process as medical supervisors. However, that said it was difficult to demonstrate value when access to patients is restricted.

This was highlighted further in that all agreed to the model for the service but implementation of the model would be for individual doctors to agree. Despite the research process and agreement with everyone regarding a way forward, it would still be within the medical consultants’ gift to approve or ignore the policy directive in practice. This powerful position could yet still prevent the service moving forward and allow the current inconsistent service to carry on.

It was thought essential within this model to have a communication debrief after each clinic with all present to discuss decisions, queries, and patient progress. Affectionately, this was known as a ‘wash up’ meeting, and in effect, this was the team working desired by all.

Whilst there was agreement that the above should be the optimal model, it was also agreed that nurses were the obvious choice to extend their role. From the previous dialogue with the pharmacist and an insider knowledge of their desire to practice in chemotherapy clinic, this was a difficult message to hear for the pharmacist present though no objections were voiced at the time.

### Open formulary

The open formulary had been contentious throughout the previous dialogue. All nurses and all pharmacists saw only benefit in the full range of drugs being available, whilst the medical staff had differing views ranging from fully supportive to ‘madness’! This poles apart view was explained to the group that evoked strong reaction.

*I think given the extreme, well I think its extreme, response, regulatory response, we have seen to the isolated insanity of Shipman it is bizarre to permit a huge body of people who have been subject to substantially less pharmacologic training, the authority to prescribe those drugs*—Doctor.

In addition, a nurse stated

I feel insulted by that view and to think anyone would suggest I might be cavalier and start managing conditions I know nothing about is unbelievable. I am a professional and have my code of conduct to guide me.

This was a difficult issue to resolve, as the nurses and pharmacists all believed they would only prescribe within their competence, as would the medical staff, yet creating a formal boundary as in a local formulary was seen as professionally abhorrent and sectarian.

*Why should we be treated any differently? Medical staff are not asked to work to a formulary, why should we. I will be accountable*—Nurse.

The above two quotes illustrate the view of all the nurses present but are interesting as they represent a changed view from the nurse focus group when their own construction was agreed. At that point, a year earlier, nurses had no objection to a local formulary in order to advance practice. Now, all nurses present resented the restrictions imposed whilst still being clear they would only prescribe within their boundaries.

### Multidisciplinary team attendance

The MDT is a multidisciplinary team meeting where all involved in the care of an individual attend to discuss the treatment plan and make decisions regarding the care of every patient. They are cancer site specific, and, generally, patients are discussed only once prior to beginning chemotherapy. In order to be involved in prescribing chemotherapy, MDT attendance was seen as vital for one particular doctor and agreement could not be reached through the medical focus group. This doctor was not present at the consensus meeting but had asked to meet after the meeting to add in his thoughts. The doctor in question was clear to say, it was not his view that he would necessarily lack confidence in a nurse-led service but he wanted to avoid anyone of his team working in isolation. The nurses and pharmacists did not have a strong view about this and wanted to be part of a team which the concurrent model above facilitates. They did not see benefit in attending the MDT.

It also became clear that the medical staff present had a little knowledge of what NMP activity took place outside their own clinic. Indeed, this lack of awareness of other NMP activity was cited by the author as one of the reasons for the ad hoc nature of the service. In hindsight, correcting the lack of awareness was clearly within our gift.

Working in isolation was a concern for all and in particular the main concern of the doctor who described non-attendance at MDT a ‘deal breaker’. It was suggested by the researcher that the practice of ensuring a ‘wash up’ meeting described above and the consistency of the same NMP nurse in each clinic might help allay concerns at not being a member of the MDT. The medical staff present thought the MDT and the ‘wash up’ meeting served very different purposes but both prevented working in isolation. Describing their own experience of MDTs those present felt, there was a little point to MDT attendance as at the point the decision is made to refer to chemotherapy the patient has yet to be seen by anyone from cancer care, and therefore their suitability for chemotherapy is unknown.

Clearly, no one present could articulate a positive view in attending the MDT, therefore a follow-up discussion with the doctor who held the view that MDT attendance was vital helped clarify his position. He conceded his view was unique and he reiterated his point that those involved in treatment must be part of the team but also recognised the view of others.

### Training

A summary document of training requirements for NMP was circulated prior to the consensus meeting as it was clearly recognised through phase 1 and 2 medical colleagues were unclear about the programme. Questions were asked regarding the governance arrangements within the Trust which reassured those present. The medical staff asked about the medical supervisor role and were reassured that they had the opportunity to work with trainees who would potentially be prescribing chemotherapy. In addition, the group was informed about the single competency framework in development for all prescribing professionals, which offered some reassurance [60].

It was agreed by all involved that NMPs should have an annual assessment of competence. Whilst this is not currently required for medical staff, the same reaction of horror from the nurses and pharmacists was not present for this initiative as for the local formulary. All agreed that it was good clinical practice in a changing health care.

## Discussion

Important to this research was that the process should generate change. One of the concerns was that despite this research process and prolonged engagement with stakeholders of the service, the practice setting will remain the same. This is put by one of critics of FGE who states that ‘Consensus does not equal commitment’ [61, p. 446].

Medical leadership within a team context is standard in chemotherapy, and nurses and pharmacists need to work within this culture. As stated by Buckley *et al* [15], the future development of any NMP is clearly dependant on well-developed relationships with medical consultants without which the service will remain static. Alongside this, nurses (and pharmacists) need to be very clear what core values underpin practice and should look to ways to demonstrate their contribution in a meaningful way. In this way, the doctor/nurse and doctor/ pharmacist substitution model can be avoided. As intimated by Goswell and Siefers [43] and Crew [44], maximizing the benefits of a flexible workforce will only occur if there is transparency about the different professions contribution in a valuable and sustainable way. This is no easy task, as despite strengthening the standards of training for NMPs, some doctors still have concerns and misunderstandings about this role [24, 62]. This view though is counterbalanced with extremely favourable outcomes from further studies in NMP [24, 25, 63]. However, whilst medicine may be leading, others are not powerless. Working alongside medical colleagues and proactively demonstrating the value of NMP in the chemotherapy setting to patients, doctors, nurses, pharmacists, managers, and the service itself should help to shape chemotherapy services of the future. Turner *et al* [64] agree and state the very best way to engage with others is to work alongside and demonstrate the value in real life.

The NCAG report is just one part of the continued implementation of the Cancer Reform Strategy [5]. It contains important recommendations that require many different teams to work together to improve the service, particularly around safety, quality, and the provision of chemotherapy. It clearly recommends nurse-led chemotherapy services and this is embraced by nurses in local practice. Yet, the evidence based on which to base this recommendation is limited. This study adds to the growing body of knowledge regarding NMP in general but in particular the acute sector and chemotherapy. This study offers the only study related to NMP of chemotherapy and one of the few in the acute sector.

Several recommendations evolve from this study:

(a) examine your local service and determine the best model for practice;
(b) develop close working relationships with medical colleagues;
(c) avoid isolation and develop clear communication channels ;
(d) be clear with colleagues your scope and boundaries of practice;
(e) develop robust services valuing your professional contribution.

For all chemotherapy services, the current focus on safety and service redesign have never been more prominent, and all chemotherapy services should seize the opportunities this focus will bring [5]. The UK Oncology Nursing Society [65] has developed a position statement to guideline practitioners in developing nurse led chemotherapy services [66]. There are nine recommendations, including the following, that enhance the findings of this study;

(a) It must not be a replacement for, or duplication of, the medical clinic.
(b) It will be underpinned by the added value of nursing practice, such as holistic patient focus and support for families and carers.
(c) Nurses should never work in isolation and there should be robust support systems.
(d) Those providing the service should have recognised qualifications and extensive experience in chemotherapy.
(e) Services will need to demonstrate effectiveness in terms of patient care and value for money.
(f) Excellent communication skills are essential.
(g) Nurse prescribing will become a gold standard in nurse-led chemotherapy review.

In agreement with the Fitzsimmons study [35], this study revealed much misunderstanding about NMP. There remains a need to continue to educate stakeholders about the potential of NMP and importantly what it does not offer. The transparency achieved through this research process will need to be further developed and maintained to avoid a repetition of misunderstandings and myth. Whilst agreement about the open formulary was eventually settled, it remains increasingly important to demonstrate the scope of practice. This transparency includes publication and distribution of annual audits of NMP practice and illustrative case studies. In addition, the developing nursing metrics for chemotherapy will be need to be embedded into practice [67]. This will require the NMPs to demonstrate strong clinical leadership and a commitment and openness to sharing the details of their practice for critique by others. In doing this, the nurse positions herself into further developing a relationship with the oncologist and this is likely to be key to the success of any NMP clinic. It also supports the best UKON’s best practice framework of demonstrating clinical effectiveness and value for money [66].

For the future, it is important to learn from the past. NMP may be an element of under graduate training for nurses and pharmacists in the next few years, and it is important to engage with all health professionals now to foster debate and break down historical barriers [68]. It will be important for NMPs to keep the local setting abreast of further developments in the journey of NMPs to avoid any repetition of resistance and myth. A major step forward has been the development of a single competency framework for all prescribers, which should help to alleviate concerns around disparity of training [69]. It would appear the NMP movement is now well established with positive evaluations and a growing confidence within all professions. For others who are struggling with implementation, there may be an opportunity to discuss the agreed shared care models implemented within this study or develop a similar training approach. Chemotherapy services are known to be disparate and there just may be some comfort in this study that nurses are not alone in their challenges to develop practice.

## Conclusion

This study is the first of its kind in a chemotherapy setting. It offers an insight into the working of the MDT in relation to NMP. It establishes the concerns and issues of the professional stakeholders as well as areas of concurrence. It charts the complexities and difficulties faced when shifting responsibilities of professionals as is required in contemporary practice. It has used a novel approach to engage with stakeholders from within the day to day practices of the clinic and developed a framework for monitoring agreed progress. The process itself facilitated an educative forum for all and further developed relationships. The study illuminated the key features necessary to maximise success of NMP in chemotherapy clinics and captures the importance of good working relationships. Whilst different models of nurse-led practice will emerge, fundamental and core to services is the need for good team working, established and effective communication strategies, and perhaps most importantly avoiding isolation in practice. Multidisciplinary working is the mantra of cancer services and this study confirmed this. However, this study additionally reinforced that any evaluation takes place within pre-existing political contexts and in particular medical leadership. Not all medical colleagues agreed with or wanted NMP for their patients highlighting difficulties of developing new models of working within a resisting culture. Medical leadership should be acknowledged and strategies developed to work within this framework to ensure the unique contribution of nursing is valued and seen. Nurses must now rise to the challenge and demonstrate clearly the added value of nursing to NMP in practice. Insights from this study raise the need for further research and exploration of the usefulness and acceptability of NMP in chemotherapy. Underpinning all should be the desire to maintain, develop, and articulate the unique contribution of nursing. This articulation informed by the gathering of quantifiable evidence in the form of metrics will help managers and others gain resources for education and sustainable services.

## Limitations

Limitations for this study include changing behaviour whilst being observed. This was not widespread and prolonged engagement is a counterargument, as others have found sustained changes in behaviour unlikely [70]. Additionally, this study relied on the ability of some doctors to articulate and comment on issues they have not experienced themselves in practice.

## Figures and Tables

**Table 1.** The differences between independent and supplementary prescribing.

Independent prescribing	Supplementary prescribing
‘Prescribing by a practitioner (e.g., doctor, dentist, nurse, pharmacist) responsible and accountable for the assessment of patients with undiagnosed or diagnosed conditions, and for decisions about the clinical management required, including prescribing’. [4, p. 2]	‘A voluntary partnership between the responsible prescriber and a supplementary prescriber, to implement an agreed patient-specific clinical management plan, with the patients agreement, particularly but not only in relation to prescribing for a specific non-acute medical condition or health need affecting the patient’. [23, p. 3]

**Table 2.** Data collection techniques at each phase of the study with purpose and identification of sample.

	Technique	Purpose	Sample
Phase 1	Individual interviews using semi-structured format	To illicit individual view	Once identified, the stakeholder was purposively approached.
Phase 2	Uni-professional focus groups	To attempt to gain a view of a particular professional group view	Participants from phase 1 were used to sample for phase 2. However, it was important to offer the opportunity to engage with all stakeholders, and therefore non-participation in phase 1 did not preclude joining discussions at phase 2.
Phase 3	Cross-professional focus group	To attempt to develop a consensus on the way forward for the service agreed by all professionals	Participation at phase 3 was about representing the stakeholder group view and negotiating contentious points with other professionals. Participants were drawn from those previously taking part in the study.

**Table 3.** Participants at each stage of the study.

Participants	Nurses	Doctors	Pharmacists	Managers	Total
Phase 3 (multi-professional focus group)	3	3	1	0	7
Phase 2 (uni-professional focus groups)	5	2 (+2 provided written comments)	2	2	11
Phase 1 (individual interviews)	5	12	4	4	25

**Table 4.** Data analysis process.

Data analysis process	Action	Product
Preliminary analysis	Involves reading and reflecting on the transcript followed by a critique of its contents line by line to try to make sense of ‘what’s going on’.	The result of this stage is broad codes or initial groups.
Thematic analysis	Builds on the preliminary analysis. Re-examine the codes created at preliminary analysis, identify those that could merge or were repetitive, and then narrow to pertinent qualitative data [71, 72].	The result of this stage was the beginning of categories or themes as a way to organise and manage the data.
Determination of claims concerns and issues	Themes were examined for claims, concerns, and issues, and these were tested through emerging constructions. The database was now quite large, and constant cross check of the themes and codes was required to ensure the data ‘fits’ the code.	The result of stage 3 was themes or constructions that represent the viewpoint of the stakeholder group and are presented as claims, concerns, and issues.
